# Metastatic Small Cell Carcinoma of the Bladder Complicated by Paraneoplastic Acute Thrombocytopenia

**DOI:** 10.7759/cureus.63480

**Published:** 2024-06-29

**Authors:** Ao Li, Luc Farnan, Craig Mulhall, Narangoda Shyamali

**Affiliations:** 1 General Medicine, Bundaberg Hospital, Bundaberg, AUS; 2 Oncology, Bundaberg Hospital, Bundaberg, AUS

**Keywords:** thrombocytopenia, metastatic bladder cancer, bladder tumour, cytotoxic chemotherapy, rural hospital, paraneoplastic syndromes, high-grade neuroendocrine tumor, small cell bladder carcinoma

## Abstract

Small cell carcinoma of the bladder is an extremely rare and aggressive disease with poor overall survival, as it is often diagnosed in later stages. Similarly, paraneoplastic thrombocytopenia is also a rare phenomenon infrequently described in the literature. Given its rarity but responsiveness to chemotherapy, awareness of atypical presentations helps facilitate appropriate treatment.

A 76-year-old gentleman was admitted to an Australian regional hospital from a small remote hospital with complaints of five months of anorexia, lethargy, weight loss, and new-onset pleuritic chest pain with a past medical history of prostatomegaly and a distant history of localised seminoma treated with surgical resection and radiotherapy alone. Physical examination revealed new rapid atrial fibrillation and mild hypoxia alongside right upper quadrant tenderness and fullness. The patient underwent pleural drainage, cytology, and computed tomography, was subsequently diagnosed with small cell carcinoma of the bladder, and rapidly developed isolated thrombocytopenia that improved with inpatient chemotherapy with carboplatin/etoposide. He was eventually discharged home after a lengthy admission. On follow-up, he had cycle 2 of treatment as an outpatient before undergoing palliative treatment at the patient’s small remote hospital.

This highlights the importance of both prompt recognition and treatment of rapidly growing small cell carcinomas when they first present atypically with uncharacteristic paraneoplastic syndromes to reduce morbidity and mortality.

## Introduction

Bladder cancer is the ninth most common cancer worldwide [1], and small cell carcinoma of the bladder is an incredibly rare neuroendocrine subtype comprising less than 1% of all urinary bladder tumours [2]. Similar to urothelial cancers of the bladder, patients are mostly male, with a mean ratio of 5:1 and a mean age of diagnosis around 67 years [3]. Despite the similarity in symptoms with other bladder carcinomas with haematuria, dysuria and frequency, small cell carcinomas present later in staging at the time of diagnosis and have a significantly reduced overall survival [1].

Thrombocytopenia in solid tumour malignancies is a relatively common occurrence due to marrow suppressive chemotherapy regiments or radiotherapy, and more rarely, direct tumour marrow involvement, disseminated intravascular coagulation, or drug-induced immune thrombocytopenia [4]. Paraneoplastic autoimmune-mediated thrombocytopenia is even rarer but a well-described phenomenon in the literature [5].

This case study presents a rare case of a metastatic small cell carcinoma of the bladder with acute thrombocytopenia that preceded any marrow suppressive chemotherapy in a regional Australian hospital.

## Case presentation

A 76-year-old, Caucasian, non-smoking male was admitted to our regional secondary centre from a rural site with complaints of left-sided, pleuritic, sub-thoracic pain and new-onset rapid atrial fibrillation. He had a past medical history of prostatomegaly and associated obstructive uropathy, as well as distant testicular seminoma with surgical resection and radiotherapy alone when he was 26 years old.

**Figure 1:**
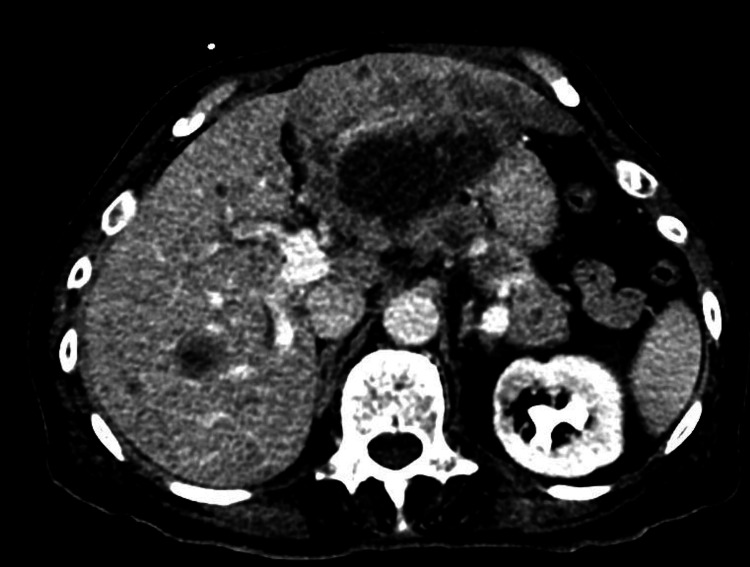
Axial CT images of the patient’s liver showing multiple metastases and the initial planned outpatient site of biopsy prior to admission CT = computed tomography

**Figure 2:**
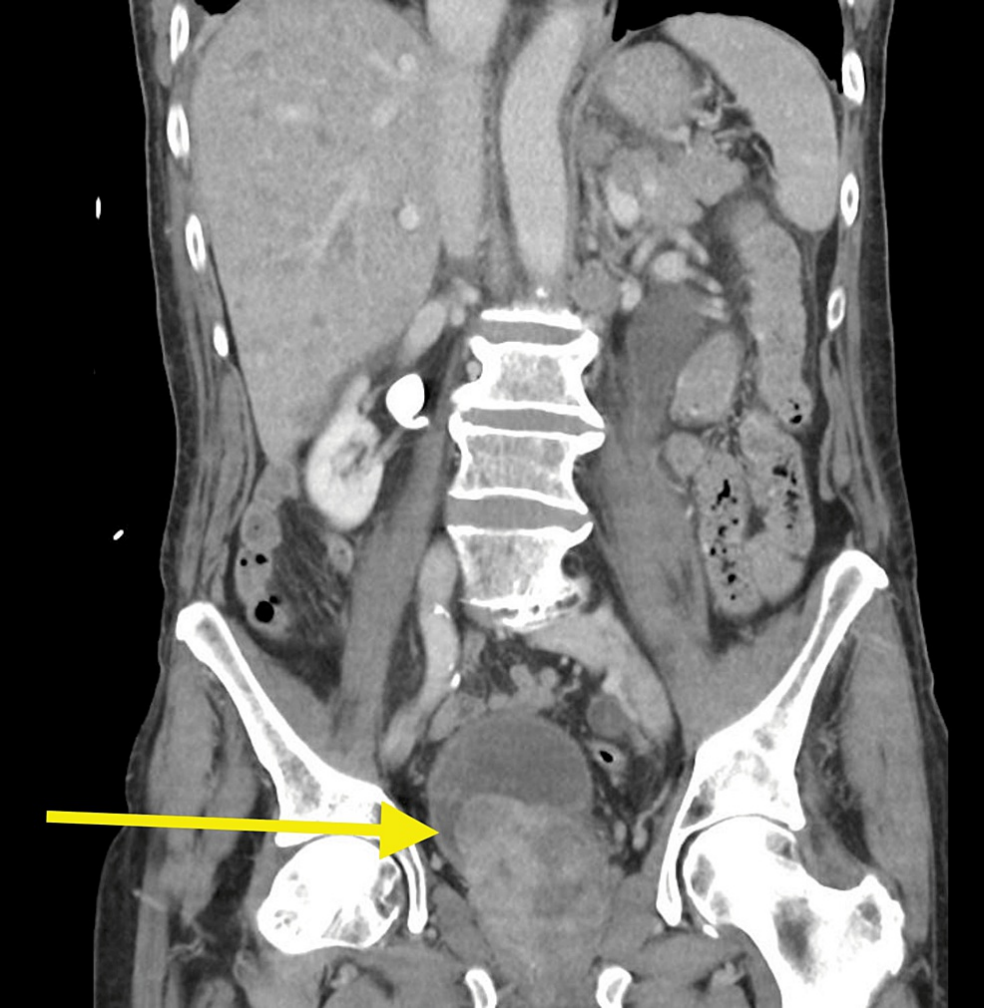
Coronal CT images of the patient’s polypous bladder mass and likely primary site of malignancy CT = computed tomography

**Figure 3:**
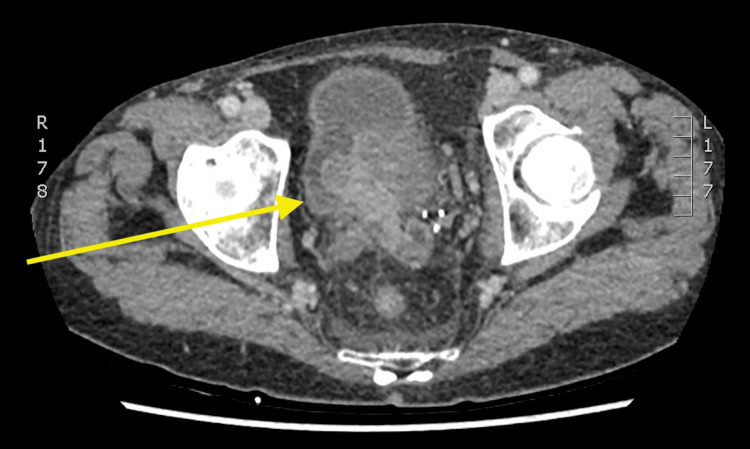
Axial CT images of the patient’s polypous bladder mass and likely primary site of malignancy CT = computed tomography

The patient was previously planned for an outpatient liver biopsy only two weeks prior to admission after a computed tomography scan had revealed an enlarged liver that was heterogeneous with multiple nodules (Figure 1) and a polypous 33x16 mm large mass lesion within the urinary bladder with proximal dilation of the entire left ureter with grade 3 obstruction (Figures 2, 3).

The patient revealed a five-month history of lethargy, anorexia and unintentional weight loss ever since admission to the hospital where he was catheterized for presumed obstructive uropathy secondary to benign prostate hyperplasia that resolved with the initiation of tamsulosin/dutasteride. On physical examination, he was found to be in new atrial fibrillation with a fluctuating heart rate of 110-150, mildly hypoxic requiring 2 litres of nasal prongs to saturate adequately, as well as exquisite left upper quadrant tenderness and fullness but no peritonism.

**Figure 4:**
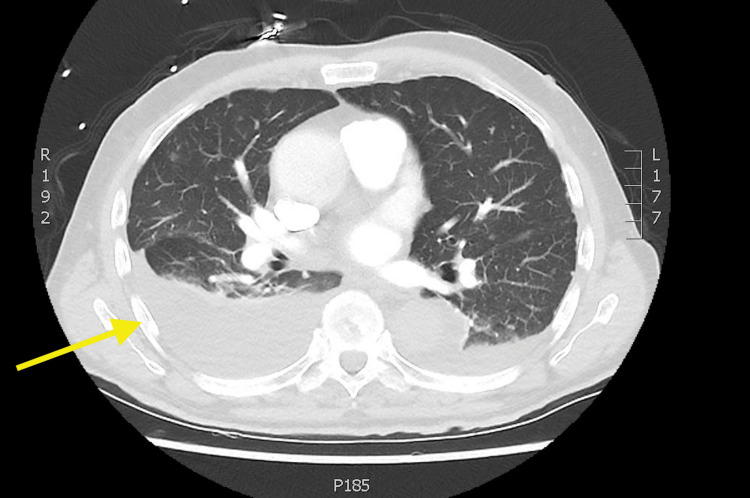
Axial CT images showing bilateral pleural effusions right worse than left CT = computed tomography

**Figure 5:**
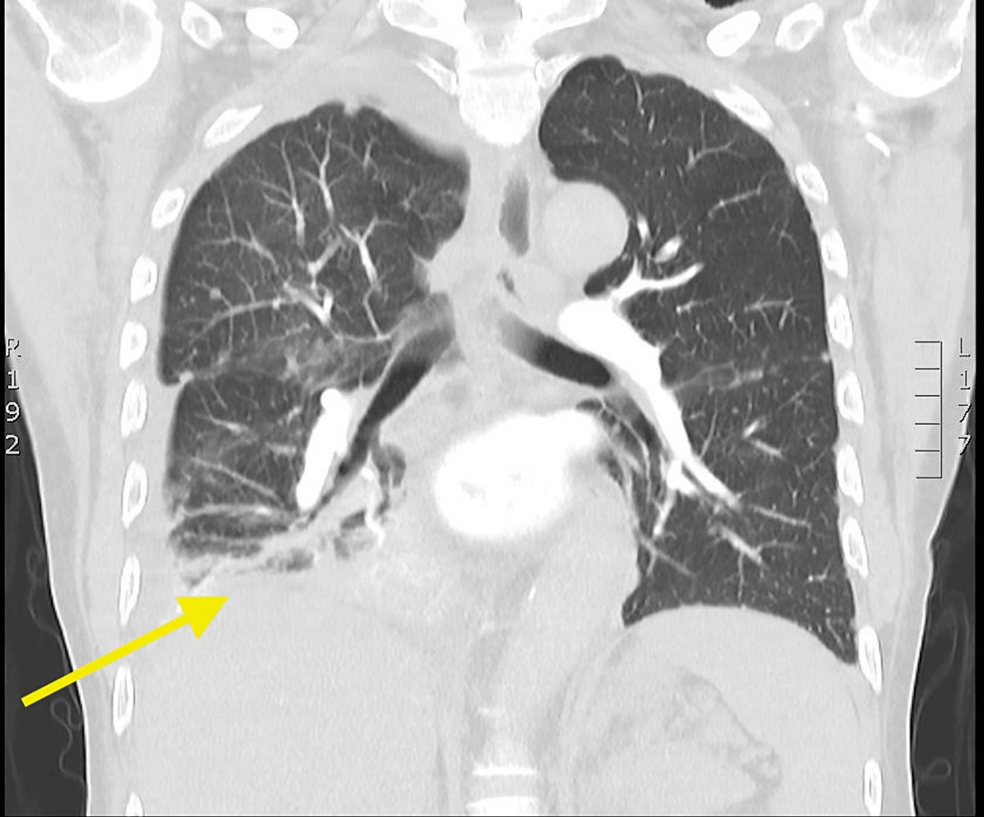
Coronal CT images of right-sided pleural effusion with small military metastases in the lung CT = computed tomography

**Figure 6:**
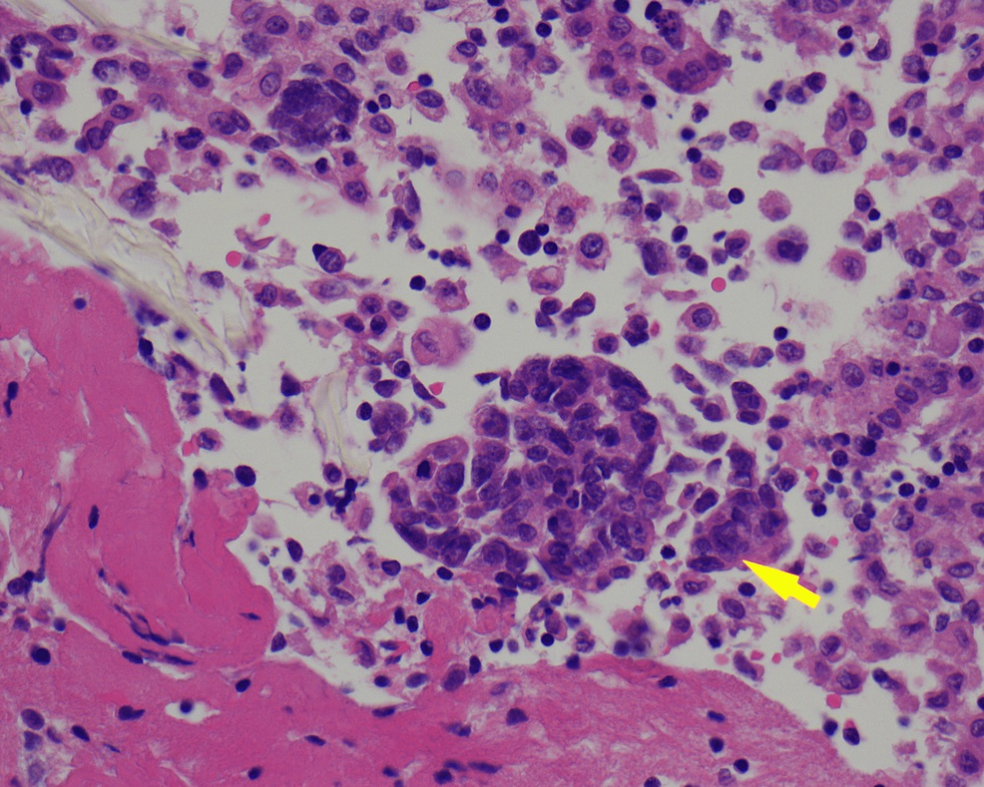
Hematoxylin and eosin stain x400 of pleural fluid showing small clusters of malignant cells as marked by the yellow arrow

**Figure 7:**
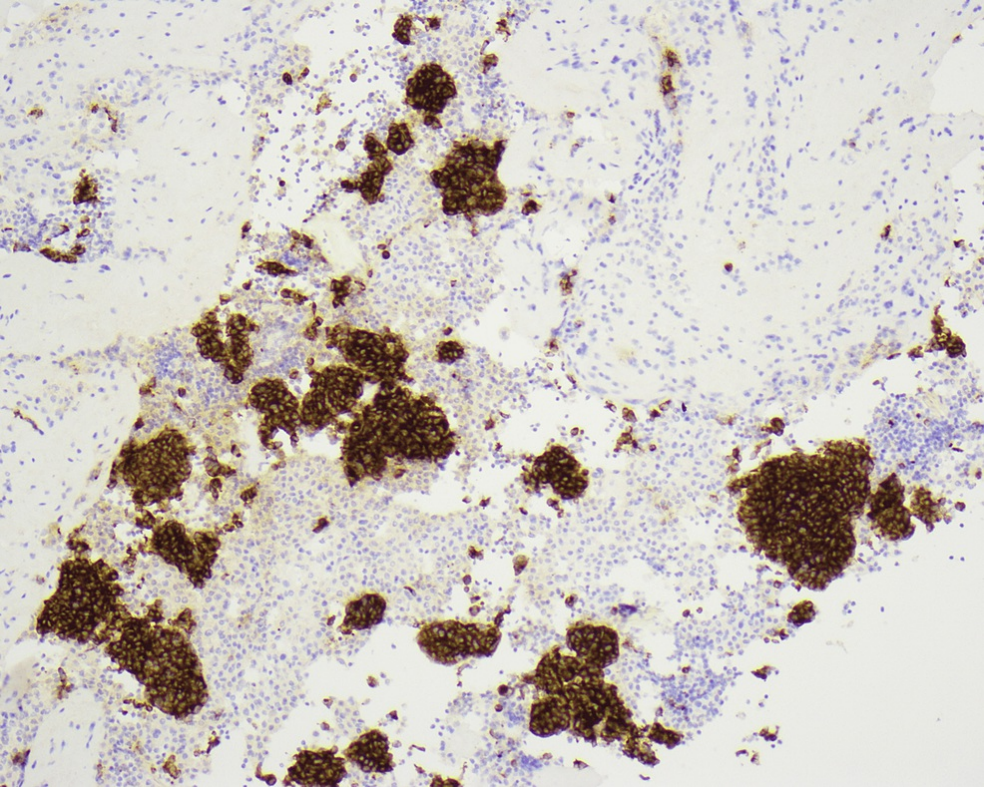
Synaptophysin staining x100 – strong staining of cytoplasm of malignant neuroendocrine cells in pleural exudate

On admission, the patient underwent a further contrast CT of the entire body, including a CT pulmonary angiography that was negative for a pulmonary embolism but revealed a large right pleural effusion with 6 cm subsegmental consolidation in the posterior aspect of the right lower lobe and a small 2 cm left-sided pleural effusion along with miliary lung metastases throughout both lobes (Figures 4, 5). There was also retroperitoneal and coeliac lymphadenopathy, but no other large masses were suspicious for alternative primaries. Ultrasound-guided chest drainage of his right-sided pleural effusion was conducted and the cytology later revealed small clusters of malignant cells with scanty cytoplasm and nuclear moulding positive for TTF1, CD56, and CK7 with a Ki67 positivity of >60%. Negative for S100, CK20, WT1, CK5/6, gata3, and p63. All of this was consistent with a grade 3 poorly differentiated neuroendocrine small cell tumour (Figures 6, 7).

**Figure 8:**
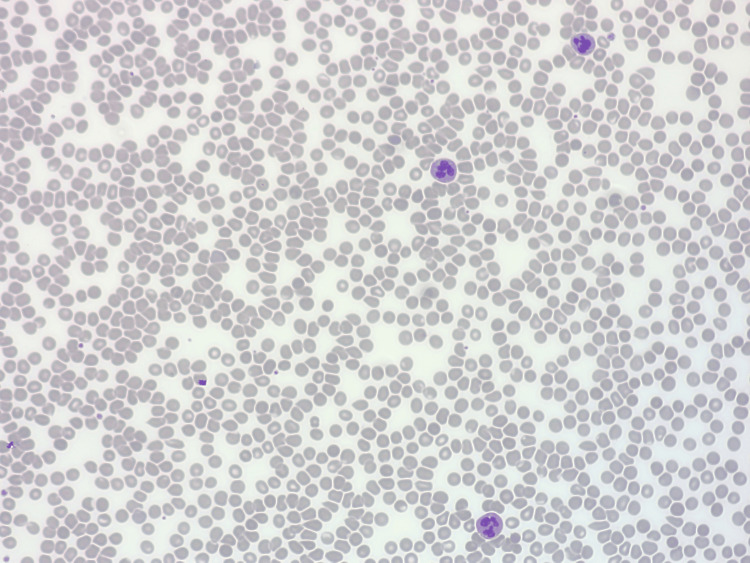
Blood film with nil significant red cell fragmentation and platelet anisocytosis consistent with marked reduced platelet counts

The patient developed rapid thrombocytopenia from platelets of 166 x109/L on admission to 87 x109/L on day 5 of admission, then 43 x109/L the day after, before rapidly declining to a nadir of 9 x109/L on day 9. Other cell lines were within normal lab limits without any significant decrease. Blood film showed nil significant red cell fragmentation with only platelet anisocytosis present (Figure 8).

Heparin-induced thrombocytopenia was initially considered given that the patient was treated with therapeutic-dose enoxaparin for pulmonary embolism by the small rural hospital prior to its exclusion. Confounding antibiotics, such as amoxicillin/clavulanic acid, were also ceased. Platelet Factor 4-Ab assay sent on day 6 of admission returned negative and subsequent haematological screens including coagulation profile, fibrinogen, D-dimer and blood films were also negative for disseminated intravascular coagulation and thrombotic microangiopathy. At this time, intravenous immunoglobulin was commenced on day 7 of admission for presumed immune-mediated thrombocytopenia alongside steroids. However, the patient’s platelets failed to respond to this as well as maintain any sustained increment post platelet transfusion; therefore, the impression was that the primary cancer was driving both consumptive and immune-mediated destruction of the platelets in the patient.

Given the aggressiveness and cytological findings, the inability to get a primary bladder tissue biopsy given thrombocytopenia and haemodynamic instability and a lack of urological services on site, the patient was discussed at a multi-disciplinary team meeting involving medical oncology and urology via telehealth and decision made to offer palliative chemotherapy. The patient’s Eastern Cooperative Oncology Group (ECOG) performance status pre-admission was 0 but was 2 at the time of discussion. The patient was started on full-dose intravenous carboplatin AUC 5 day 1 and etoposide 100 mg/m^2^ days 1-3 as an inpatient on day 13 of admission.

The patient’s clinical course was complicated by severe deconditioning as well as febrile neutropenia, mild tumour lysis requiring only intravenous fluids, and blood transfusion requirements due to chemotherapy-induced pancytopenia. Initial platelets at the start of treatment were 12 x10 9/L, which further declined with expected marrow toxicity. However, after 13 days post-chemotherapy, the patient’s platelets began to increment without transfusion support above 52 x109/L. The patient underwent rehabilitation and was eventually discharged home rurally after 31 days of admission with a normal platelet count of 235 x109/L with plans to receive outpatient day-unit chemotherapy for his ongoing treatment.

He returned to the oncology outpatients to have cycle 2 of chemotherapy with full-dose carboplatin and etoposide. Unfortunately, 14 days post cycle 2, he was admitted to his local hospital with neutropenic sepsis. The patient, however, declined transfer for further assessment and treatment and opted for a palliative approach dying 33 days later.

## Discussion

Small cell carcinoma of the bladder is rare, aggressive, and presents in later stages. They also compromise less than 1% of all urinary bladder tumours [2]. Unlike pulmonary small cell carcinoma, small cell carcinoma of the bladder is staged with the traditional TNM staging system. On diagnosis, more than 95% are T2 or higher, with 26.1% in one study metastatic on diagnosis [1]. Median survival for localised disease is 21 months [6] and with metastatic disease, the median survival is seven to thirteen months [7].

Direct bladder tissue diagnosis would have been preferred over pleural fluid cytology, as most case reports have transurethral biopsies [3,8], and even on cystoscopy, small cell and transitional cell carcinomas cannot be differentiated [2]. However, this was difficult being in a regional site without access to urological services on-site, and with distant urban centres' reluctance for transfers given the patient’s acute thrombocytopenia increasing the risk of further biopsies [9] as well as haemodynamic instability with ongoing hypoxia. As such, ultimately, a time-critical decision was made with a multidisciplinary team meeting to diagnose small cell carcinoma of the bladder based on imaging and cytology alone, especially given the lack of any large lung or other primary malignancy on imaging. This decision was made to facilitate immediate chemotherapy especially given doublet platinum and etoposide chemotherapy for small cell carcinoma of the bladder is similar to the treatment of small cell carcinoma of the lung [7] as well as small cell carcinoma of unknown origin [10]. Being located remotely likely had an impact on the patient's late presentation with metastatic disease, which is consistent with other studies on this topic for other cancers [11].

Our case also presents acute isolated thrombocytopenia, which is a common issue in malignancy, especially in solid organ tumours where it is commonly a side effect of the myelotoxic chemotherapy regimens or due to direct tumour invasion into bone marrow [9]. Interestingly, our patient’s thrombocytopenia precedes any such treatment and while tumour invasion is a possibility, there were no obvious signs of bone metastases on any imaging and there have also been rare case reports of bone marrow involvement of metastatic urothelial cancer and very few with small cell bladder carcinoma [12].

The patient was initially evaluated for heparin-induced thrombocytopenia (HITS) given the temporal relationship with therapeutic enoxaparin especially since studies have suggested that HITS is more common in oncological patients compared to baseline populations [9]. Other notable common causes in oncological patients were abruptly ruled out such as disseminated intravascular coagulation and thrombotic microangiopathy [13]. Confounding drug-induced thrombocytopenia was also addressed although amoxicillin/clavulanic acid-induced thrombocytopenia is also incredibly rare with very few case reports [14]. Given that the patient’s platelet count failed to recover a week after ceasing the antibiotics, the fact it recovered post-chemotherapy and the failure to maintain increments with transfusions potentially implies a cancer-mediated paraneoplastic process. Paraneoplastic autoimmune thrombocytopenia has also been described rarely in the literature for solid organ tumours but these usually involve lung and breast cancers [7], and there have been some case studies for paraneoplastic thrombocytopenia secondary to small cell carcinomas of the lungs [15]. However, none were described in small cell bladder cancer.

There is no consensus on the best treatment guidelines for small carcinoma of the bladder disease and the most common regimen is derived from the treatment of lung small cell carcinoma. For metastatic disease, one study used alternating regiments of ifosfamide/doxorubicin with cisplatin/etoposide for 12 patients, with a median overall survival of 13 months [7]. Furthermore, novel immunotherapy agents, such as pembrolizumab, have only been described in case reports [16], and more studies are needed in this field. Unlike small cell carcinoma of the lung, there is no established role for prophylactic cranial radiation [17]; however, radiation can be used for local control or for palliating metastatic bone pain. Unfortunately, our patient only had a survival of two months post-diagnosis. Indeed, this reinforces that the rarity and rapid progression of the disease limits in-depth research.

## Conclusions

Although incredibly rare, prompt recognition of atypical presentations of small cell carcinoma especially with paraneoplastic syndromes, is important to expedite appropriate treatment. With such treatment, the chemo-sensitivity of these tumours often leads to a quick response and, hopefully, can reduce morbidity and delay mortality.
